# Common disease signatures from gene expression analysis in Huntington’s disease human blood and brain

**DOI:** 10.1186/s13023-016-0475-2

**Published:** 2016-08-01

**Authors:** Eleni Mina, Willeke van Roon-Mom, Kristina Hettne, Erik van Zwet, Jelle Goeman, Christian Neri, Peter A.C. ’t Hoen, Barend Mons, Marco Roos

**Affiliations:** 1 Department of Human Genetics, Leiden University Medical Center, PO Box 9600, Leiden, 2300 RC The Netherlands; 2 Department of Medical Statistics and Bioinformatics, Leiden University Medical Center, PO Box 9600, Leiden, 2300 RC The Netherlands; 3 CNRS, UMR 8256, Laboratory of Neuronal Cell Biology and Pathology, Institute of Biology Paris-Seine, 9 quai Saint Bernard, Paris, 75005 France; 4 Sorbonnes Universités, University Pierre and Marie Curie (UPMC) Univ Paris 06, 4 place Jussieu, Paris, 75005 France

**Keywords:** Huntington’s disease, Blood, Biomarker discovery, Concept profile analysis, WGCNA

## Abstract

**Background:**

Huntington’s disease (HD) is a devastating brain disorder with no effective treatment or cure available. The scarcity of brain tissue makes it hard to study changes in the brain and impossible to perform longitudinal studies. However, peripheral pathology in HD suggests that it is possible to study the disease using peripheral tissue as a monitoring tool for disease progression and/or efficacy of novel therapies. In this study, we investigated if blood can be used to monitor disease severity and progression in brain. Since previous attempts using only gene expression proved unsuccessful, we compared blood and brain Huntington’s disease signatures in a functional context.

**Methods:**

Microarray HD gene expression profiles from three brain regions were compared to the transcriptome of HD blood generated by next generation sequencing. The comparison was performed with a combination of weighted gene co-expression network analysis and literature based functional analysis (Concept Profile Analysis). Uniquely, our comparison of blood and brain datasets was not based on (the very limited) gene overlap but on the similarity between the gene annotations in four different semantic categories: “biological process”, “cellular component”, “molecular function” and “disease or syndrome”.

**Results:**

We identified signatures in HD blood reflecting a broad pathophysiological spectrum, including alterations in the immune response, sphingolipid biosynthetic processes, lipid transport, cell signaling, protein modification, spliceosome, RNA splicing, vesicle transport, cell signaling and synaptic transmission. Part of this spectrum was reminiscent of the brain pathology. The HD signatures in caudate nucleus and BA4 exhibited the highest similarity with blood, irrespective of the category of semantic annotations used. BA9 exhibited an intermediate similarity, while cerebellum had the least similarity. We present two signatures that were shared between blood and brain: immune response and spinocerebellar ataxias.

**Conclusions:**

Our results demonstrate that HD blood exhibits dysregulation that is similar to brain at a functional level, but not necessarily at the level of individual genes. We report two common signatures that can be used to monitor the pathology in brain of HD patients in a non-invasive manner. Our results are an exemplar of how signals in blood data can be used to represent brain disorders. Our methodology can be used to study disease specific signatures in diseases where heterogeneous tissues are involved in the pathology.

**Electronic supplementary material:**

The online version of this article (doi:10.1186/s13023-016-0475-2) contains supplementary material, which is available to authorized users.

## Background

Huntington’s disease (HD) is a devastating disease that is inherited in an autosomal dominant manner. The genetic cause of the disease is a CAG repeat expansion in the coding region of the huntingtin gene (*HTT*). This is translated to an expanded stretch of glutamine amino acids in the huntingtin protein (HTT) and this mutant protein is the main cause of neuropathology in HD. While extensive research has been done since 1993, when the genetic cause of the disease was discovered [1], there is still no cure for this disease nor an effective treatment.

Clinical and imaging biomarkers have been developed that measure the disease state and progression [2]. Nevertheless, these biomarkers can be expensive and often cannot monitor changes before onset of clinical symptoms. To develop an intervention that starts before disease onset it is important to have biomarkers that can accurately measure changes between controls and HD patients before symptoms first arise. To date, promising clinical trials targeting the mutant protein are under development and robust as well as reliable biomarkers are essential to advance these novel therapeutic strategies into the clinic.

Although the main pathology of HD is found in the brain, human brain tissue cannot be used to measure molecular biomarkers to monitor disease state and progression in living patients. However, due to the ubiquitous expression of the mutant protein, the HD phenotype is not limited to the brain. Symptoms such as weight loss, skeletal muscle wasting and cardiac failure, point out an altogether complex pathology that involves many tissues [3, 4]. This opens the opportunity to investigate HD related pathology in more accessible tissues that can be obtained in a non-invasive manner. Transcriptional dysregulation is a prominent feature of the disease. Expression profiling studies in brain have shown that in the caudate nucleus 21 % (9763) of the probe sets demonstrated significant differential expression [5]. Investigating gene expression changes in peripheral tissue can provide new insights that can lead to the development of new therapies and biomarkers to monitor disease progression.

Using post mortem brain tissue can however introduce biases when studying disease mechanisms due to non-disease specific effects of post mortem interval and specific agonal conditions such as coma, hypoxia and seizures [6]. Several studies have focused on the analysis of blood using microarray technology, to study the pathology in HD. However, HD-specific gene expression changes are less pronounced in blood and it has proven difficult to validate them across studies [7, 8]. For example, Borovecki et al. analyzed global changes in mRNA expression in the blood samples of HD patients, compared with controls and identified a set of 12 genes that were able to clearly distinguish controls and patients with HD [9]. Although this work was highly promising, to this date their results have proven difficult to replicate.

More promising biomarkers emerged with the advances in next-generation sequencing. Mastrokolias et al. identified a HD signature that included 40 genes that were previously reported in at least one HD gene expression study with the same direction in expression change [10].

However, Cai et al. [11] showed that little preservation occurs in mean expression levels between the brain and blood. It is however possible that signals are preserved at levels beyond gene expression. For instance, Chuang et al. pointed out that subnetwork markers in a protein-network-based approach were significantly more reproducible than individual gene markers in two different cancer cohorts [12].

The most robust HD disease signature based on transcriptomics data to be used for drug development and disease progression biomarkers should be present in both blood and brain [13, 14]. Because the blood signature is derived from non-neuronal tissue and the brain signature is masked by non-HD related processes such as hypoxia, the shared signature is likely the most informative from a mechanistic and therapeutic point of view. We used a systems biology approach that combines Weighted Gene Co-expression Network Analysis (WGCNA) [15, 16] and literature mining technology [17, 18] to assess the similarity between brain and blood tissue using previously published gene expression studies in brain and blood [5, 10]. We prioritized signatures that were shared between blood and brain tissue at a systems level, based on mechanisms that involve multiple genes and proteins. In general, genes that are part of the same mechanism, exhibit similar expression changes. At the mechanistic level we can compare signature signals from post-mortem HD brain tissue and blood to provide novel biomarkers that can be measured in blood to monitor the brain pathology in living patients. Such an approach offers many advantages and can also be useful for other neurodegenerative disorders. Apart from the non-invasive nature of blood sampling, it is also cost effective and widely available. This can lead to the development of more standardized tests and offer more robust measurements.

## Methods

### Blood dataset

The blood dataset used in this work consists of transcriptomics data obtained by next generation sequencing (NGS) from whole blood. The dataset consists of 33 controls, 27 presymptomatic mutation carriers and 64 symptomatic mutation carriers. The phenotypes associated with this dataset that we used in this analysis were carrier status, CAG repeat length, and two clinical scores namely motor score and Total Functional Capacity (TFC) score. The TFC score is inversely correlated to the motor score and the CAG repeat, and it reflects the disease severity with lower numbers indicating higher severity of the disease. The carrier status was assigned ’0’ for controls and ’1’ for HD carriers. The CAG repeat values ranged between 17 and 53, covering both controls and HD samples. The motor score of the control subjects ranged between 0 and 11 and between 0 and 107 for the HD subjects. The TFC score for the control subjects ranged between 11 and 13 and for the HD subjects between 0 and 13.

We used the processed blood data set that is publicly available in the NCBI Gene Expression Omnibus (GEO) database under accession number GSE51799. Details for the dataset can be found in the original publication [10].

We removed the presymptomatic samples from the blood samples because there were only 3 presymptomatic samples in the brain study. We also removed transcripts with low tag count (less than 94). The dataset was normalized using the calcNormFactors (TMM method) from the edgeR bioconductor package for RNA-Seq [19]. In addition, the dataset was transformed using the method described in [20].

### Brain dataset

The HD human brain data used in this analysis were obtained from the public repository NCBI Gene Expression Omnibus, entry GSE3790. The data set was originally created and analyzed by Hodges et al. [5]. It contains 44 HD gene positive cases and 36 age and sex matched controls. They analyzed three different brain regions, caudate nucleus, frontal cortex (BA4 and BA9 regions) and cerebellum, with an Affymetrix Microarray GeneChip (Human Genome U133A and U133B). In addition, they classified the HD cases based on Vonsattel grade of disease pathology (scale = 0 –4). We used the processed data from the Human Genome array U133A to construct the WGCNA network. The dataset was log2 transformed.

### Application of weighted gene co-expression network analysis (WGCNA)

The transformed blood and brain datasets were used to construct the weighted gene co-expression networks. We used the original WGCNA algorithm as described in [15].

The parameters were chosen based on the assumption of a scale-free topology of the co-expression network, according to WGCNA guidelines, and also in order to make modules from each network of comparable sizes. Other parameters followed default settings that are based on earlier investigations of the WGCNA method by Horvath et al. [15, 16]. The soft threshold for each network was: caudate: 9, BA4: 3, BA9: 5, cerebellum: 5, blood: 9. The parameter minModuleSize was assigned to 15 for all networks. We performed module identification using the dynamic tree cut algorithm [21]. The parameters for the module identification were chosen as to avoid creating many large modules. We chose method = “hybrid” for all networks, deepSplit = 2 for all brain networks and deepSplit = 0 for the blood network. Changing the deepSplit parameter that results in larger or smaller modules appeared to have minimal effect on the composition of genes and annotations of modules (data not shown). The parameter cutHeight was chosen as cutHeight = 0.999 for all brain networks and cutHeight = 0.995 for the blood network. For the blood network we chose MEDissThres = 0.30 for merging modules whose expression profile was similar. In order to keep the module sizes of the brain networks comparable to the blood network the MEDissThres parameter for all four brain networks was chosen to be MEDissThres = 0.0001. In total we identified 34 modules in the blood dataset consisting of a median of 66 genes (mean:233). In the brain dataset we identified in total 55 modules in the caudate nucleus, 67 in BA4, 118 in BA9 and 81 in cerebellum. Details on the association of modules per network with the disease phenotypes can be found in Additional file 1.

Module size per network in the brain datasets were for the caudate median: 105.0, mean: 335, for the cerebellum median: 75, mean: 275.1, for the BA4 median: 67, mean: 332.6 and for the BA9 mean: 51.5, median: 188. The association of modules with the disease phenotypes was performed with an implementation of the R’s standard cor function in the WGCNA package. The correlation method that was used was Pearson correlation.

### Concept profile analysis

We performed Concept profile analysis (CPA) which was previously described in [17, 18]. In short, CPA is based on comparing concept profiles that were mined from the literature. To construct a concept profile, PubMed abstracts are indexed by Peregrine (https://trac.nbic.nl/data-mining/), using a thesaurus of biomedical and chemical concepts that has been prepared in-house for text mining [22, 23]. For every concept in the thesaurus that is associated to at least five PubMed records, a concept profile is created. A concept profile is a vector containing all concepts related to the main concept (by direct co-occurrence in PubMed abstracts). Each relation is weighted by the symmetric uncertainty coefficient. Concept profiles are matched with each other to identify similarities via their shared concepts (indirect relations). Any distance measure can be used for this matching such as the mutual information, inner product, cosine, angle, Euclidean distance or Pearson’s correlation. The CPA Web Services that we used for our analysis use the inner product measure.

### Module annotation - parameters and semantic types

The modules were annotated with concepts of a certain semantic type using the Taverna [24, 25] workflow which is publicly available from the myExperiment repository http://www.myexperiment.org/workflows/3921.html. This workflow orchestrates a series of CPA web services for module annotation which use literature that was mined up to July 2012. We used a selection of 4 predefined concept sets from our local database of concept profiles. A concept set is a collection of concept profiles related to a particular semantic type (e.g. biological processes). The concept sets that we used in our analysis were: biological processes (ID:5), cellular component (ID:3), molecular function(ID:4) and disease or syndrome (ID:82). The first three concepts sets are based on the Gene Ontology (GO). Gene Ontology (GO) terms are often given as words or phrases that are infrequently found in normal texts. To still provide broad coverage of GO terms, we previously developed a procedure specifically for GO [17]. In short, the GO concept profiles were created based on the literature (PubMed identifiers) provided in the GO database itself. That includes literature based on all gene-GO term associations and GO-terms without an explicit association with genes (version 2012-07-14, direct download link: http://archive.geneontology.org/lite/2012-07-14/go_20120714-assocdb.rdf-xml.gz). The disease or syndrome concept set provides information about diseases or syndromes that currently exist in the UMLS [26] and OMIM databases [27], by using information from the complete literature. The top 20 annotations (based on the sum of their similarity scores with all of the genes in a module) from each concept set was used to annotate each module. The annotation of modules was performed by matching the concept profiles of the genes in the module with each of the concept sets. To subsequently define the similarity between a pair of modules (one from brain and one from blood), we counted the number of overlapping annotations between each module pair.

### Randomization approach and significance of the modules

The following algorithm was applied separately for each of the comparisons between the datasets and for each semantic annotation category separately. Suppose we have identified n modules *A*_1_,*A*_2_,…,*A*_*n*_ in blood and m modules *B*_1_,*B*_2_,…,*B*_*m*_ in one of the brain regions. We use CPA to annotate each module and compute similarity scores *S*_*ij*_ for all pairs (*A*_*i*_,*B*_*j*_), based on the number of overlapping annotations. To assess the significance of these similarity scores, we use a permutation-based approach as follows. For *k*=1,2,…100, we generate sets of random modules *A*_1*k*_,*A*_2*k*_,…,*A*_*nk*_ and *B*_1*k*_,*B*_2*k*_,…,*B*_*mk*_ of the same size as the original modules *A*_1_,*A*_2_,…,*A*_*n*_ and *B*_1_,*B*_2_,…,*B*_*m*_. We compute similarity scores *S*_*ijkl*_ for each pair (*A*_*ik*_,*B*_*jl*_). On the basis of these 10,000 permutation-scores, we use Westfall and Young’s minP method [28] to compute the significance of each module pair (Ai,Bj). Note that this method aims to control the familywise error rate (FWER) by adjusting for the fact that we make *n*×*m* comparisons. As explained in [29], the fact that the scores are discrete causes the Westfall-Young method to be conservative. To counter this, we have decided to set the significance level at 10 %. We do point out that to reach this level of significance, the observed similarity score should be the most extreme among all permutations. Also, driven by biological interpretation of module pairs that are associated with sexual differentiation that had a large number of overlapping annotations and number of overlapping genes we decided to include module pairs up to a significance level of 50 %. These module pairs are indicated in Additional file 2 with dashed lines.

## Results

### Workflow to identify HD modules in blood and common functional signatures between blood and brain

**Fig. 1:**
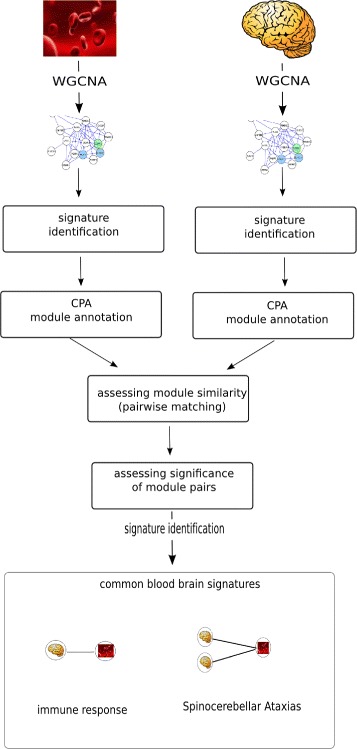
Workflow for the identification of common signatures between blood and brain tissue. The blood and brain datasets are transformed into gene co-expression networks using Weighted Gene Co-expression Network Analysis (WGCNA). The networks are represented by modules, groups of highly co-expressed genes. Modules significantly correlated with HD are identified. The modules are annotated using Concept Profile Analysis (CPA), using four semantic annotation categories i.e. Biological Processes, Cellular Component, Molecular Function and Disease or Syndrome. Next, the similarity between the annotated modules from each network is assessed (for each semantic annotation category) based on the total number of overlapping annotations between each blood-brain module pair (pairwise matching). The significance of each module pair is assessed by repeating the entire analysis using randomly composed modules of the same gene size as the original ones. The random distribution was used to assign a significance value for each module pair. At the bottom of the figure, the module pairs that cross our threshold (*P* *v* *a* *l* *u* *e*<0.05) which compose our two blood-brain signatures

In order to identify common Huntington disease-specific signatures in brain and blood, we created groups of co-expressed genes (modules) using weighted gene co-expression network analysis (WGCNA). We first applied WGCNA to the blood and brain dataset and identified modules that were associated with the disease phenotype. We then used Concept Profile Analysis (CPA) technology to annotate each module separately with different semantic annotation categories and assess the similarity between the different networks (blood versus the four brain regions) based on the overlap of the module annotations (Fig. 1). The annotations were based on four semantic categories: biological processes, cellular component, molecular function and disease or syndrome. The categories correspond to the main branches in the gene ontology as a natural way to distinguish between different views on biological function [30]. The gene enrichment analysis that CPA performs differs from the classical GO gene enrichment analysis because CPA annotates genes by mining biomedical literature, allowing the identification of more specific GO categories as previously demonstrated in [30], while the GO annotations are manually curated.

### Disease signatures identified in the blood data set

**Fig. 2:**
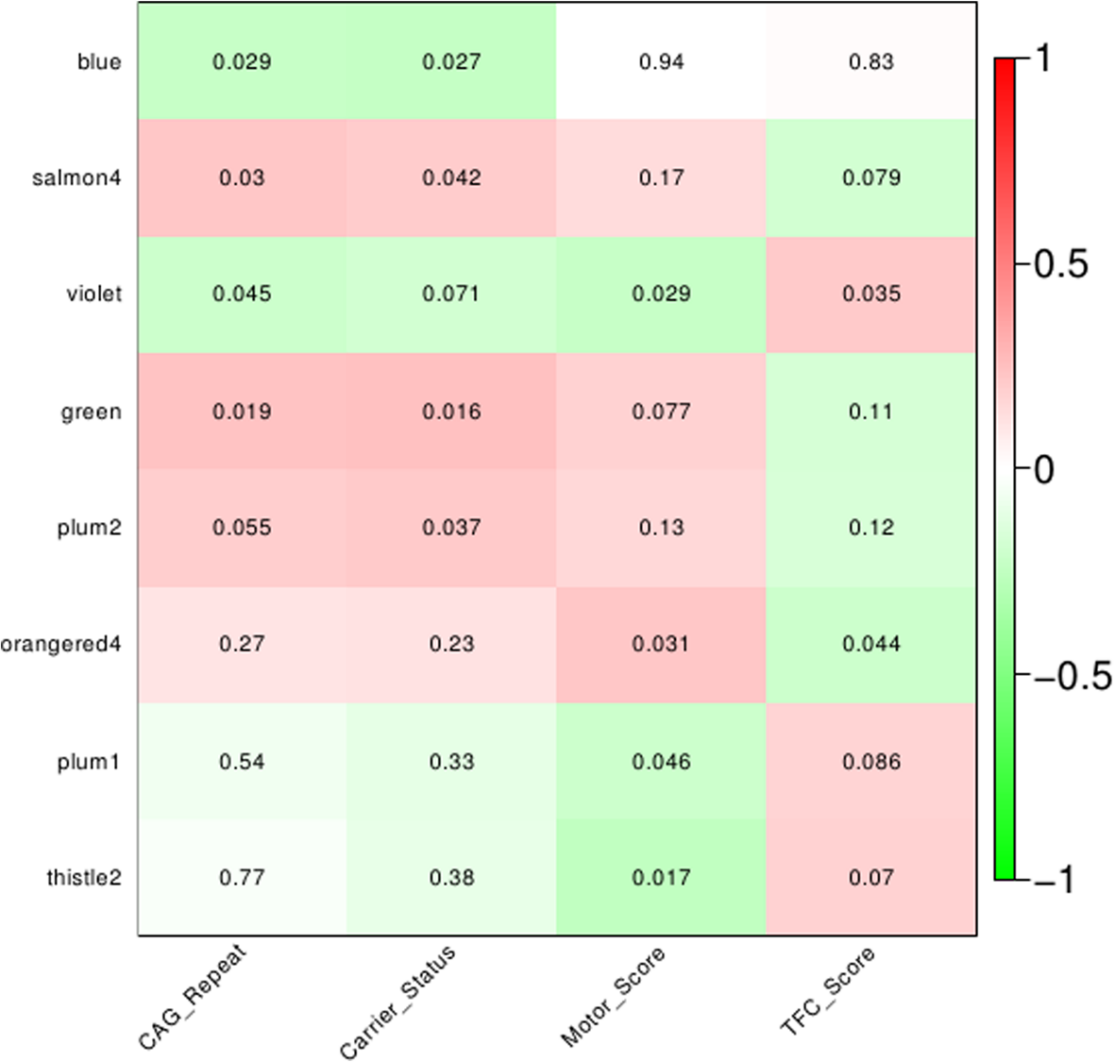
Significantly correlated blood modules with 4 different disease phenotypes. Each module was assigned a different color name, cf standard WGCNA. Significant modules were identified by correlating the module eigengenes (first principle component of the summary of gene expression profiles within a module) to the disease phenotypes, i.e. carrier status, CAG repeat, motor score and Total Functional Capacity (TFC) score, a measure for disease severity. On the y-axis: the top 8 correlated modules, x-axis: the disease phenotypes. The numbers represent the *P* *v* *a* *l* *u* *e* for the correlation per module. *Green*: negative correlation, *red*: positive correlation. The intensity of the color depicts the strength of the correlation
- Cell cycle and protein transport (green module) were positively correlated with motor score and CAG repeat. Recent studies in mouse models of HD suggest that abnormalities in the cell cycle may contribute to the HD pathogenesis [32, 33].
- Sphingolipid biosynthetic process (thistle2 module) was positively correlated with TFC score and negatively correlated with motor score. Sphingolipids are essential for brain function and recently they were reported as candidate biomarkers in Alzheimer’s disease based on evidence that sphinglolipid levels are associated with cerebrospinal fluid (CSF) amyloid *β*42 concentration [34]. In addition, an independent study reported sphingolipids being associated with restoration of motor behavior in HD mice [35].
- Cell signaling and synaptic transmission (orangered4 module) were positively correlated with motor score and negatively with TFC score. The HAP1 gene is included in this module that encodes for the huntingtin associated protein 1 which directly binds to huntingtin [36].
- Phospholipid transport and dephosphorylation, and RNA splicing (salmon4 module) were positively correlated with CAG repeat and carrier status. Phospholipids have been proposed as targets for therapeutic intervention in HD, because of the finding that mutant huntingtin is able to interact with phospholipids in membranes [37], with different affinity and preference for specific phospholipids, depending on the polyglutamine expansion [38].

For our functional analysis of HD signals in blood data, we used our blood network modules that were identified by WGCNA. We identified in total 8 modules that were correlated with the disease phenotype from a total number of 34 modules (Fig. 2). Each module was annotated using CPA and the most representative annotations comprised the signature of that particular module. The extended list of the blood modules and their associated annotations can be found in Additional file 3 and more detailed information about gene composition and annotations per module in Additional files 4 and 5. Here, we describe the top 5 signatures.

- Immune response (violet module) was positively correlated with TFC score and negatively correlated with CAG repeat length and motor score. Inflammation can be detected in monocytes from presymptomatic patients [31] and a gene expression inflammation signature was also reported by Mastrokolias et al. [10].

The number of genes that were part of the 8 modules that were correlated with HD accounted for 8.72 % of the total number of genes detected in the blood experiment. In contrast, only 1.03 % of genes was identified as differentially expressed using classical differential gene expression (DGE) analysis [10]. Investigating this further, we find an overlap of twelve genes between the differentially expressed genes from the DGE analysis and the genes that were part of the significantly correlated modules. Ten out of the 62 genes from the inflammation module overlapped with this common set of 12 genes, suggesting a strong disease effect on the expression of these genes.

### WGCNA in the brain dataset

We also applied WGCNA to the four brain regions separately to identify modules that are correlated with the disease phenotype. WGCNA has been applied before in the same brain dataset [39–41] but we had to reanalyze this data to obtain module sizes that were comparable with the modules from the blood dataset.

We found the highest number of modules associated with the HD phenotype in the caudate nucleus (37 modules). Cerebellum and BA4 follow with 29 and 23 modules respectively (Additional files 1 and 6). BA9 exhibited poor association with HD since none of the BA9 modules were significantly correlated with the disease state. However, a large number of modules exhibited a strong association with the disease staging phenotype (Additional files 1 and 6).

Because the semantic annotations of the modules from each brain network can be relevant for future research, we report here the processes that were prominently present in all brain regions: immune response, chromatin remodeling, histone modification, cell-cycle, myelination and cell differentiation (oligodendrocyte, axon and Schwann cells), synaptic activity, protein transport, nuclear activity, protein kinase activity and RNA splicing.

Our analysis confirmed the neurodegeneration pattern in HD as previously described in [5], and was also described by the WGCNA analysis in [39]. Overall the functional comparison of our semantic annotations with the results from [39] showed a high degree of similarity. For instance, RNA splicing, protein transport, protein modification, immune response, chromatin organization and DNA damage were identified by both analyses.

### Association of modules between different tissues - module pairing

In order to identify signatures that represent mechanisms that are common in HD blood and brain tissue we used the annotated modules from each network (blood, caudate nucleus, cerebellum, BA9 and BA4) to compare them pairwise and calculate the number of overlapping annotations for each blood-brain pair for the four semantic annotation categories separately (Fig. 1). Only significant blood-brain pairs were considered for further analysis. The significance of each module pair was assessed by repeating the entire analysis using randomly composed modules of the same number of genes, which we annotated using the same four annotation categories. A significance value was assigned to each module pair using a random distribution.

**Fig. 3:**
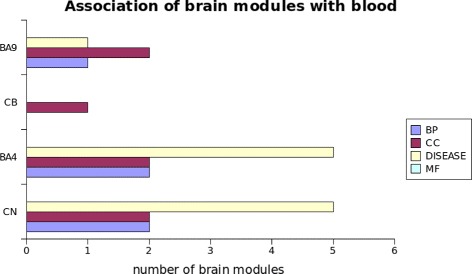
Semantic comparison between the modules of four brain regions and blood. The total number of blood-brain pairs per semantic type above our significance threshold (*F* *W* *E* *R*<=10 *%*) is depicted by each color bar. Blood exhibits the highest similarity with both the caudate and BA4. BA9 and cerebellum follow with slightly higher scores for BA9

The similarity between blood and each brain region was assessed by the total number of significant module pairs per semantic annotation category. Caudate nucleus and BA4 exhibited the highest similarity with blood (Fig. 3), in all semantic annotation categories. BA9 exhibited an intermediate similarity, while cerebellum had the least similarity with only one significant module pair in the cellular component annotation category. None of the brain regions exhibited similarity with blood based on the molecular function annotation category.

### Common signatures shared by blood and brain

**Fig. 4:**
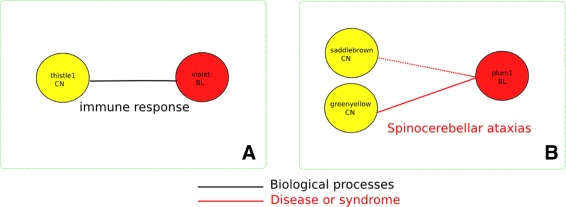
The two blood brain signatures identified by our analysis. The *yellow circles* indicate caudate nucleus modules and red color indicates blood modules. The line between two modules indicate a significant link (blood-brain pair) between modules (*F* *W* *E* *R*<=10 *%*). Dashed line indicates a module pair with marginal significance (*F* *W* *E* *R*<50 *%*). Different color lines indicate an association based on each semantic annotation. *Black*: Biological processes, *red*: Disease or Syndrome

We define a blood-brain module pair to represent a common disease signature when it meets the following criteria: 1: significance value of the blood-brain module pairs, 2: each module separately is associated with at least one of the HD phenotypes as identified by WGCNA. We named the signatures according to their most representative annotation. From the total number of associated modules between blood and brain (Fig. 3) we identified in total two common disease signatures based on these criteria (Fig. 4; Additional files 7 and 8). We named the signatures according to their most representative annotation.

Signature 1. The immune response signature was shared between blood and caudate (Fig. 4a). This signature was identified based on the biological processes annotation category. Common annotations included major histocompatibility complex location, lymphocyte activation, cytokine activity, T cell activation and adaptive immune response. The caudate nucleus module consisted mainly of HLA genes of the major histocompatibility complex class I (A, B, C, F, G).

Signature 2. The Spinocerebellar ataxias (SCAs) signature was shared between blood and the caudate nucleus (2 modules) (Fig. 4b), based on the disease or syndrome annotation category. The link that was shared between one of the caudate nucleus modules (saddlebrown; indicated with a dashed line) and blood achieved marginal significance, with FWER of 30 %. The annotations that were common in this signature were multiple forms of SCAs, from both the polyglutamine and non-polyglutamine repeat disease class (SCA types 1,2,3,6,7,8,10,12 and 17).

### Comparison with preservation and gene wise overlap

**Fig. 5:**
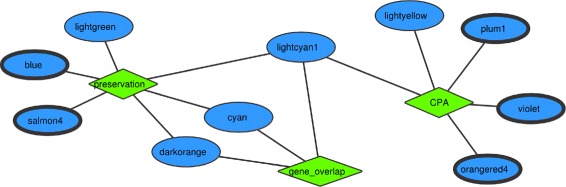
Comparison of the results between CPA in four semantic annotation categories, gene overlap counts, and module preservation. For the preservation analysis, we used the Z-summary metric, a composite preservation measure that identifies modules that are preserved in other networks based on their connectivity and density statistics. Significant module pairs for the gene wise overlap were computed the same way as for the annotation overlap using CPA (Methods). The figure shows the blood modules that were significantly associated with the caudate nucleus network. Each different method is depicted with a *green diamond*. The *oval shapes with thick black lines* represent modules significantly correlated with the disease phenotype. Only one module is shared by all three approaches (lightcyan1), which is not significantly correlated with the disease phenotype. The overall overlap between the methods is small

To investigate how our method compares to other methods, we compared the results of the blood - caudate nucleus semantic analysis to gene overlap counts between blood and caudate nucleus modules, and to the preservation statistics method from the WGCNA itself (Fig. 5). The gene overlap analysis identified in total 3 blood modules exhibiting significant gene overlap with the caudate nucleus modules, of which none was significantly correlated with the disease phenotype. The preservation from the WGCNA identified in total 6 blood modules that were preserved in the caudate nucleus (Zcore values: 2<*Zcore*<10) of which two were significantly correlated with HD (blue and salmon4). Our methodology, using CPA, identified in total 5 blood modules associated with the caudate nucleus modules; three of which were significantly correlated with the disease (violet, plum1, orangered4) (Fig. 5). This shows that our analysis is comparable to other well-established methods and can produce results that are rather complementary for the identification of common disease signatures between blood and brain.

## Discussion

In this paper we used WGCNA and literature information to identify modules in blood that are associated with the HD phenotype and to identify disease signatures that are shared between blood and brain. To our knowledge, this is the first time that the similarity between blood and brain tissue was successfully assessed based on a combination of WGCNA and literature information. WGCNA was used in order to group genes of the same tissue that are co-expressed (modules), while literature information (CPA) was used to annotate and evaluate the similarity between modules from different tissues at a functional level.

In summary, we identified 8 HD-specific modules in blood and two distinct signatures that are shared between blood and brain. The HD-specific modules in blood were associated with immune response, sphingolipid biosynthetic process, lipid transport, cell cycle, protein modification, spliceosome, RNA splicing, vesicle transport, cell signaling and synaptic transmission. This analysis points to mechanisms that are affected in HD. Some were already known to be implicated in the brain pathology, but their role in blood has not been elucidated yet [31, 32, 42, 43].

The scarcity of HD brain tissue has driven research to use blood to identify biomarkers that can be used to study disease state and disease progression that are most clearly observed in the brain. In previous studies the similarity between blood and brain was assessed based on the conservation of gene expression patterns only [8–10]. Such assessments are usually very difficult, because blood and brain are two inherently different tissues composed of very different cell types. Nevertheless, in our study, we discovered signatures that are based on a functional similarity between blood and brain. We argue that the same function may partially be executed by different gene products in different cell types, also considering that our current knowledge may have different gaps across cell types. At the functional level the active units are not merely the genes, but cells and organs. Cells of different types that play a role in the execution of a biological function express different genes that are thus associated with that function. It is therefore a fair assumption that when comparing between cell types, we should look beyond the level of individual genes. For instance, microglia and macrophages both participate actively in the immune response but different sets of genes are expressed in microglia or macrophages [44]. Another example where two different gene products carry out the same function is hemoglobin and myoglobin. Both genes are associated with transport of oxygen, but one is expressed in red blood cells and the other in muscle [45–47]. Therefore, signatures based on co-expression and functional annotation are more likely to represent disease-specific mechanisms. We speculate that a common signature at the functional level is more robust, which makes it attractive to monitor disease progression or the efficacy of a particular treatment. The blood-brain signature allows us to focus on a specific part of the blood signal to monitor the HD-affected brain.

Our findings suggest that mechanisms associated with inflammatory response and SCA are important mechanisms that are shared between blood and brain in HD (Fig. 4). The inflammation response may be an important component of HD pathology that contributes to the neuropathological damage. This finding supports previous detection of abnormal activation of immune response in HD patients [31]. In addition, this signature links the well-established neuroinflammation signature in brain to a parallel inflammatory response in blood, triggered upon expression of the mutant huntingtin. The same signature was also identified by Horvath and colleagues in their study of the preservation of brain modules in two large blood cohorts of healthy individuals [11]. Although they were unable to identify full module preservation, they found that a subset of the genes that was preserved was functionally enriched in, among others, infectious disease and infection mechanisms. Both analyses point to an immune response mechanism as a shared channel between blood and brain. Although the mechanisms preserved in those datasets came only from healthy individuals, we conclude that this preserved signal is also specific for HD.

In addition, we showed that blood exhibits similarities with brain based on different criteria (Fig. 3). These criteria reflect similarities on a functional level i.e. biological processes, cellular component, molecular function, and functions associated with the same disease or syndrome. The disease or syndrome annotation led us to the identification of the SCA signature (Fig. 4b). The association of blood modules with brain disorders is by itself an interesting topic for further research. A signature based on commonality in disease or syndrome annotations would have been difficult to identify by approaches that only focus on gene expression or traditional annotation schemes (e.g. GO based annotation). Genes that were part of this signature on the brain side were the *TCF4, ATN1, PPP2R2B, ATXN10* and *ATXN3*, which are associated with neurodegenerative and developmental disorders such as Pitt-Hopkins Syndrome [48], Dentatorubral pallidoluysian atrophy [49], SCA12 [50], SCA10 [51] and SCA3 [52]. On the blood side of this signature were among others the *PPP2R2B* gene associated with SCA12, *CAPNS2*, which was recently found to play a beneficial role against polyglutamine toxicity [53], and *SETBP1* which is associated with the Schinzel-Giedion syndrome [54, 55].

The comparison of the results of our methodology with those obtained by the preservation statistics from WGCNA, and those from assessing the gene overlap between blood and brain, shows that the three methodologies are complementary. The overlap between the three methods was very small (Fig. 5) indicating that they identify similarities based on different criteria. In fact, the signature that was identified by all methods was not associated with the HD phenotype, but with sexual differentiation. The modules in this signature are strongly co-expressed and composed mainly of genes expressed on the X and Y chromosome. The identification of this signature served also as a control for testing the validity of each method. Depending on the hypothesis that drives one’s analysis, one or a combination of these methods could be used. Our method has the advantage that for identifying similarity we solely look at similarity at a functional level, i.e. without bias in terms of overlapping genes or similarity in expression patterns between the two tissues.

### Additional disease relevant signatures

In addition to the aforementioned signatures that were selected by the most strict criteria, we identified two additional module pairs based on less stringent criteria that we considered interesting for HD. The first criterion that we used in this analysis was that a module pair needed to achieve a significance level of 10 % of FWER. As explained in the Methods section, we define a “gray” significance level of up to 50 %, based on the significance levels that we observed for the sexual differentiation modules that served as internal control. The second criterion was that each module in particular needed to be significantly correlated (*Pvalue*<0.05) with at least one of the disease phenotypes. Detailed information about these common signatures can be found in Additional files 9 and 10.

The synapse signature was shared between blood and the caudate nucleus based on the cellular component annotation category (Additional file 2A). The caudate nucleus module (indicated with a dashed circle) was marginally associated with the disease staging phenotype (*Pvalue*=0.074; Additional file 1) while the blood module was associated with motor and TFC score. Common annotations in this signature were associated with synapse activity and dendrites. Albeit the absence of cells with synapses in the blood, the annotation of the blood module with concepts like “synapse” hints at the presence of gene products with a function in the neuronal synapse and an alternative function in the blood, which may be used as a surrogate marker for the effect of disease on neuronal transmission.

The genes that contribute the most to the synaptic activity annotation are *NOS1, HAP1, GRIK2, HTR6*. Literature supports that at least three of them are directly implicated in HD [36, 56, 57]. In fact, HAP1 was one of the first proteins that were described to interact with huntingtin [36]. The genes encode ubiquitously expressed proteins with a known function in brain, but their role in blood remains elusive. *GRIK2* was proposed as a candidate biomarker in Chronic Fatigue Syndrome (CFS) after it was detected in the peripheral blood of CFS patients as differentially expressed [58]. *NOS1* was also associated in studies as being able to regulate blood pressure [59].

In addition, 7 out of 18 members of this module (MTRNR2L1, LAPTM5, QRICH1, MOAP1, AKR1B1, NOS1, ZNF260) were found to be indirectly associated with huntingtin through the interaction with the UBC protein that is known to interact with huntingtin [60–65].

Finally, cell-cell signaling (*NOS1, HAP1, GRIK2, HTR6*), ion transport (*HAP1, UNC80, KCNAB1*), cell death, and apoptosis (*LAPTM5, MTRNR2L1, MOAP1, QRICH1*) were secondary annotations associated with this blood module. The majority of the genes in this module encode ubiquitously expressed proteins that are likely to have a catalytic role in the HD blood, similar to their effect in brain. The synaptic signature can potentially be of great value for monitoring synaptic activity in brain by monitoring these genes in HD blood.

The vesicle trafficking and protein transport signature was shared between blood and BA4 based on the cellular component annotation category (Additional file 2B). In this signature, the blood module was marginally associated with the CAG repeat phenotype and also this module pair was identified with a significance level of 50 % of FWER. The annotations that were shared in this signature were related to endosomes, trans-golgi network and clathrins. This signature was also identified by Horvath et al. as a preserved mechanism between blood and brain in healthy individuals [11].

Both the synaptic activity and vesicle transport signatures have been long implicated in HD. Huntingtin is expressed in the cytoplasm where it directly interacts with a number of proteins involved in synaptic activity and vesicle transport [66]. In addition, huntingtin has been previously described as a protein that acts as a mediator in information trafficking between different cell compartments by interacting with other proteins [67]. Recent evidence suggests that synapse loss and other features of the disease that involve the CNS can be treated by targeting organs outside the CNS [68]. The blood modules involved in these signatures that link the blood with the brain pathology could become subject for further research to confirm whether symptoms of the disease can be treated by targeting factors that associate with these modules [68].

Although the blood-brain signatures that we identified are promising, there are certain limitations in our methodology that can be improved in follow up studies. Considering that similarity was assessed by overlap in annotations, future studies can extend the power of the method by using the hierarchy of an ontology to assign a score to annotations that are subclasses of the same function. Furthermore, the results from the computational analysis can be corroborated by further validation on additional data and by new experiments in the laboratory. The genetic predictability of HD allows for testing those signatures in carriers of the gene mutation both in mouse and humans, even before the first symptoms arise. We are currently investigating the analysis of data from human blood samples that were collected from the same subjects, but 4 years later as an accurate way of determining whether these signatures have changed over the time and whether they correlate with the progression of the disease. Testing these signatures in mouse models of HD to follow the efficiency of novel disease treatment strategies would also be beneficial for using and optimizing blood as a diagnostic and monitoring tool.

## Conclusion

In summary, we present functional HD-specific co-expression signatures in blood samples that link specific processes, measured in blood, to the pathology of HD. These signatures can be used to further study changes in blood and identify biomarkers that can track disease stage and progression. In addition, we identified two common signatures that are shared between blood and multiple brain regions in Huntington’s Disease. These signatures are likely an indication of disease changes that occur in parallel between these two tissues. These results are a step towards using blood as a surrogate to study the pathology in brain in HD, by using only that part of the signal that we can link to processes known to be in common and associated with the disease. Having a peripheral measure to monitor brain pathology opens new gateways to have a more in-depth understanding at several time points of disease progression and monitor the efficacy of treatments in a non-invasive manner. Our approach is applicable to other disorders where it is not feasible to obtain data from the most affected tissues.

## Abbreviations

CPA, concept profile analysis; FWER, familywise error rate; HD, Huntington’s disease; SCA, spinocerebellar ataxia; TFC, total functional capacity; WGCNA, weighted gene coexpression analysis

## Additional files

Additional file 1 Significantly correlated modules in brain. This file contains the modules from each brain region (caudate, BA4, BA9 and cerebellum) that were associated with the disease phenotype. The numbers (*P* *v* *a* *l* *u* *e*) indicate the correlation of each module with the disease phenotype. Green: negative correlation, red: positive correlation. The intensity of the color depicts the strength of the correlation. (PDF 1341 kb)

Additional file 2 Additional disease signatures. This file contains the additional disease signatures that were identified with less stringent statistical criteria. The yellow circle indicates a caudate nucleus module, bluegrey circle indicates a module from the BA4 brain region and red color indicates blood modules. The line between two modules indicates a significant link (blood-brain pair) between modules (*F* *W* *E* *R*<=10 *%*). Dashed line indicates a module pair with marginal significance (*F* *W* *E* *R*<50 *%*) while dashed circles indicate modules with marginal significance with the disease phenotype. The green line between module pairs defines an association based on cellular component. (PNG 66.6 kb)

Additional file 3 Modules in blood significantly correlated with HD. This file contains the 8 modules that were identified in blood as being correlated to the HD phenotype. The file describes each module according to the most representative annotations per semantic category (biological processes, cellular component, molecular function, disease or syndrome). (DOC 16.5 kb)

Additional file 4 Annotations of blood modules associated with HD. In this file we include detailed information about the annotations per semantic type of each module in blood that is associated with HD. (XLS 37 kb)

Additional file 5 Gene identifiers of blood modules associated with HD. In this file we include the gene identifiers that constitute each module in blood that is associated with HD. (XLS 84 kb)

Additional file 6 Association of the brain regions with the disease phenotype. This file describes the total number of modules, per brain region, that are associated with the the two HD phenotypes; the disease state (HD or control) and disease staging (grade 0 - 5) as that was resulted by the WGCNA. (PNG 54.9 kb)

Additional file 7 Immune response signature. In this file we include detailed information about the immune response signature, including the annotations and gene identifiers of each module that participates in this signature. (XLS 15 kb)

Additional file 8 SCA signature. In this file we include detailed information about the SCA signature, including the annotations and gene identifiers of each module that participates in this signature. (XLS 25 kb)

Additional file 9 Synapse signature. In this file we include detailed information about the synapse signature, including the annotations and gene identifiers of each module that participates in this signature. (XLS 12.5 kb)

Additional file 10 Vesicle transport signature. In this file we include detailed information about the vesicle transport signature, including the annotations and gene identifiers of each module that participates in this signature. (XLS 13 kb)

## References

[1] MacDonald ME, Ambrose CM, Duyao MP, Myers RH, Lin C, Srinidhi L, Barnes G, Taylor SA, James M, Groot N, MacFarlane H, Jenkins B, Anderson MA, Wexler NS, Gusella JF, Bates GP, Baxendale S, Hummerich H, Kirby S, North M, Youngman S, Mott R, Zehetner G, Sedlacek Z, Poustka A, Frischauf AM, Lehrach H, Buckler AJ, Church D, Doucette-Stamm L, O’Donovan MC, Riba-Ramirez L, Shah M, Stanton VP, Strobel SA, Draths KM, Wales JL, Dervan P, Housman DE, Altherr M, Shiang R, Thompson L, Fielder T, Wasmuth JJ, Tagle D, Valdes J, Elmer L, Allard M, Castilla L, Swaroop M, Blanchard K, Collins FS, Snell R, Holloway T, Gillespie K, Datson N, Shaw D, Harper PS (1993). A novel gene containing a trinucleotide repeat that is expanded and unstable on huntington’s disease chromosomes. Cell.

[2] Tabrizi SJ, Scahill RI, Owen G, Durr A, Leavitt BR, Roos RA, Borowsky B, Landwehrmeyer B, Frost C, Johnson H, Craufurd D, Reilmann R, Stout JC, Langbehn DR, TRACK-HD Investigators (2013). Predictors of phenotypic progression and disease onset in premanifest and early-stage huntington’s disease in the TRACK-HD study: analysis of 36-month observational data. Lancet Neurol.

[3] van der Burg JMM, Björkqvist M, Brundin P (2009). Beyond the brain: widespread pathology in huntington’s disease. Lancet Neurol.

[4] Sassone J, Colciago C, Cislaghi G, Silani V, Ciammola A (2009). Huntington’s disease: the current state of research with peripheral tissues. Exp Neurol.

[5] Hodges A (2006). Regional and cellular gene expression changes in human huntington’s disease brain. Hum Mol Genet.

[6] Tomita H, Vawter MP, Walsh DM, Evans SJ, Choudary PV, Li J, Overman KM, Atz ME, Myers RM, Jones EG, Watson SJ, Akil H, Bunney WE (2004). Effect of agonal and postmortem factors on gene expression profile: Quality control in microarray analyses of postmortem human brain. Biol Psychiatry.

[7] Runne H, Kuhn A, Wild EJ, Pratyaksha W, Kristiansen M, Isaacs JD, Régulier E, Delorenzi M, Tabrizi SJ, Luthi-Carter R (2007). Analysis of potential transcriptomic biomarkers for huntington’s disease in peripheral blood. Proc Natl Acad Sci.

[8] Lovrecic L, Kastrin A, Kobal J, Pirtosek Z, Krainc D, Peterlin B (2009). Gene expression changes in blood as a putative biomarker for huntington’s disease. Mov Disord.

[9] Borovecki F, Lovrecic L, Zhou J, Jeong H, Then F, Rosas HD, Hersch SM, Hogarth P, Bouzou B, Jensen RV, Krainc D (2005). Genome-wide expression profiling of human blood reveals biomarkers for huntington’s disease. Proc Natl Acad Sci U S A.

[10] Mastrokolias A, Ariyurek Y, Goeman JJ, van Duijn E, Roos RA, van der Mast RC, van Ommen GB, den Dunnen JT, ’t Hoen PA, van Roon-Mom WM (2015). Huntington’s disease biomarker progression profile identified by transcriptome sequencing in peripheral blood. Eur J Hum Genet.

[11] Cai C, Langfelder P, Fuller TF, Oldham MC, Luo R, van den Berg LH, Ophoff RA, Horvath S (2010). Is human blood a good surrogate for brain tissue in transcriptional studies?. BMC Genomics.

[12] Chuang HY, Lee E, Liu YT, Lee D, Ideker T. Network-based classification of breast cancer metastasis. Mol Syst Biol. 2007;3(1). 10.1038/msb4100180 PMC2063581 17940530

[13] Dudley JT, Tibshirani R, Deshpande T, Butte AJ (2009). Disease signatures are robust across tissues and experiments. Mol Syst Biol.

[14] Liew CC, Ma J, Tang HC, Zheng R, Dempsey AA (2006). The peripheral blood transcriptome dynamically reflects system wide biology: a potential diagnostic tool. J Lab Clin Med.

[15] Langfelder P, Horvath S (2008). WGCNA: an r package for weighted correlation network analysis. BMC Bioinforma.

[16] Zhang B, Horvath S. A general framework for weighted gene co-expression network analysis. Stat Appl Genet Mol Biol. 2005;4(1). 10.2202/1544-6115.1128 16646834

[17] Jelier R, Schuemie MJ, Roes PJ, van Mulligen EM, Kors JA (2008). Literature-based concept profiles for gene annotation: The issue of weighting. Int J Med Inform.

[18] Jelier R, Schuemie MJ, Veldhoven A, Dorssers LC, Jenster G, Kors JA (2008). Anni 2.0: a multipurpose text-mining tool for the life sciences. Genome Biol.

[19] Robinson MD, McCarthy DJ, Smyth GK (2010). edgeR: a bioconductor package for differential expression analysis of digital gene expression data. Bioinformatics.

[20] Ribeca P, Sammeth M. Pitfalls of correlation coefficients applied to gene expression data. 2011.

[21] Langfelder P, Zhang B, Horvath S (2008). Defining clusters from a hierarchical cluster tree: the dynamic tree cut package for r. Bioinformatics.

[22] Hettne KM, van Mulligen EM, Schuemie MJ, Schijvenaars BJ, Kors JA (2010). Rewriting and suppressing UMLS terms for improved biomedical term identification. J Biomed Semant.

[23] Hettne KM, Stierum RH, Schuemie MJ, Hendriksen PJM, Schijvenaars BJA, Mulligen EMv, Kleinjans J, Kors JA (2009). A dictionary to identify small molecules and drugs in free text. Bioinformatics (Oxford, England).

[24] Hull D, Wolstencroft K, Stevens R, Goble C, Pocock MR, Li P, Oinn T (2006). Taverna: a tool for building and running workflows of services. Nucleic Acids Res.

[25] Wolstencroft K, Haines R, Fellows D, Williams A, Withers D, Owen S, Soiland-Reyes S, Dunlop I, Nenadic A, Fisher P, Bhagat J, Belhajjame K, Bacall F, Hardisty A, Nieva de la Hidalga A, Balcazar Vargas MP, Sufi S, Goble C (2013). The taverna workflow suite: designing and executing workflows of web services on the desktop, web or in the cloud. Nucleic Acids Res.

[26] Bodenreider O (2004). The unified medical language system (UMLS): integrating biomedical terminology. Nucleic Acids Res.

[27] Hamosh A, Scott AF, Amberger JS, Bocchini CA, McKusick VA (2005). Online mendelian inheritance in man (OMIM), a knowledgebase of human genes and genetic disorders. Nucleic Acids Res.

[28] Westfall PH, Young SS. Resampling-Based Multiple Testing: Examples and Methods for *P*-Value Adjustment. A Wiley-Interscience publication: Wiley; 1993.

[29] Goeman JJ, Solari A (2014). Multiple hypothesis testing in genomics. Stat Med.

[30] Jelier R, Goeman JJ, Hettne KM, Schuemie MJ, Dunnen JTd, Hoen PACt (2011). Literature-aided interpretation of gene expression data with the weighted global test. Brief Bioinform.

[31] Björkqvist M, Wild EJ, Thiele J, Silvestroni A, Andre R, Lahiri N, Raibon E, Lee RV, Benn CL, Soulet D, Magnusson A, Woodman B, Landles C, Pouladi MA, Hayden MR, Khalili-Shirazi A, Lowdell MW, Brundin P, Bates GP, Leavitt BR, Möller T, Tabrizi SJ (2008). A novel pathogenic pathway of immune activation detectable before clinical onset in huntington’s disease. J Exp Med.

[32] Das E, Jana NR, Bhattacharyya NP (2015). Delayed cell cycle progression in STHdh^(q111)^/hdh^(q111)^ cells, a cell model for huntington’s disease mediated by microRNA-19a, microRNA-146a and microRNA-432. MicroRNA.

[33] Liu KY, Shyu YC, Barbaro BA, Lin YT, Chern Y, Thompson LM, James Shen CK, Marsh JL (2015). Disruption of the nuclear membrane by perinuclear inclusions of mutant huntingtin causes cell-cycle re-entry and striatal cell death in mouse and cell models of huntington’s disease. Hum Mol Genet.

[34] Fonteh AN, Ormseth C, Chiang J, Cipolla M, Arakaki X, Harrington MG. Sphingolipid metabolism correlates with cerebrospinal fluid beta amyloid levels in alzheimer’s disease. PLoS ONE. 2015. 10(5). 10.1371/journal.pone.0125597 PMC4418746 25938590

[35] Di Pardo A, Maglione V, Alpaugh M, Horkey M, Atwal RS, Sassone J, Ciammola A, Steffan JS, Fouad K, Truant R, Sipione S (2012). Ganglioside GM1 induces phosphorylation of mutant huntingtin and restores normal motor behavior in huntington disease mice. Proc Natl Acad Sci U S A.

[36] Li XJ, Li SH, Sharp AH, Nucifora FC, Schilling G, Lanahan A, Worley P, Snyder SH, Ross CA (1995). A huntingtin-associated protein enriched in brain with implications for pathology. Nature.

[37] Kegel-Gleason KB (2013). Huntingtin interactions with membrane phospholipids: Strategic targets for therapeutic intervention?. J Huntington’s Dis.

[38] Kegel KB, Sapp E, Alexander J, Valencia A, Reeves P, Li X, Masso N, Sobin L, Aronin N, DiFiglia M (2009). Polyglutamine expansion in huntingtin alters its interaction with phospholipids. J Neurochem.

[39] Neueder A, Bates GP (2014). A common gene expression signature in huntington’s disease patient brain regions. BMC Med Genomics.

[40] Oldham M, Langfelder P, Horvath S (2012). Network methods for describing sample relationships in genomic datasets: application to huntington’s disease. BMC Syst Biol.

[41] Oldham MC, Konopka G, Iwamoto K, Langfelder P, Kato T, Horvath S, Geschwind DH (2008). Functional organization of the transcriptome in human brain. Nat Neurosci.

[42] Sathasivam K, Neueder A, Gipson TA, Landles C, Benjamin AC, Bondulich MK, Smith DL, Faull RLM, Roos RAC, Howland D, Detloff PJ, Housman DE, Bates GP (2013). Aberrant splicing of HTT generates the pathogenic exon 1 protein in huntington disease. Proc Natl Acad Sci U S A.

[43] Twelvetrees AE, Yuen EY, Arancibia-Carcamo IL, MacAskill AF, Rostaing P, Lumb MJ, Humbert S, Triller A, Saudou F, Yan Z, Kittler JT (2010). Deslivery of GABAARs to synapses is mediated by HAP1-KIF5 and disrupted by mutant huntingtin. Neuron.

[44] Butovsky O, Jedrychowski MP, Moore CS, Cialic R, Lanser AJ, Gabriely G, Koeglsperger T, Dake B, Wu PM, Doykan CE, Fanek Z, Liu L, Chen Z, Rothstein JD, Ransohoff RM, Gygi SP, Antel JP, Weiner HL (2014). Identification of a unique TGF- *β*-dependent molecular and functional signature in microglia. Nat Neurosci.

[45] Wittenberg BA, Wittenberg JB (1989). Transport of oxygen in muscle. Annu Rev Physiol.

[46] Ordway GA, Garry DJ (2004). Myoglobin: an essential hemoprotein in striated muscle. J Exp Biol.

[47] Adamson JW, Finch aCA (1975). Hemoglobin function, oxygen affinity, and erythropoietin. Annu Rev Physiol.

[48] Forrest MP, Hill MJ, Quantock AJ, Martin-Rendon E, Blake DJ (2014). The emerging roles of TCF4 in disease and development. Trends Mol Med.

[49] Suzuki Y, Yazawa I (2011). Pathological accumulation of atrophin-1 in dentatorubralpallidoluysian atrophy. Int J Clin Exp Pathol.

[50] O’Hearn E, Holmes SE, Margolis RL (2012). Spinocerebellar ataxia type 12. Handb Clin Neurol.

[51] Teive HAG, Munhoz RP, Arruda WO, Raskin S, Werneck LC, Ashizawa T (2011). Spinocerebellar ataxia type 10 - a review. Parkinsonism Relat Disord.

[52] Evers MM, Toonen LJA, van Roon-Mom WMC (2014). Ataxin-3 protein and RNA toxicity in spinocerebellar ataxia type 3: current insights and emerging therapeutic strategies. Mol Neurobiol.

[53] Menzies FM, Garcia-Arencibia M, Imarisio S, O’Sullivan NC, Ricketts T, Kent BA, Rao MV, Lam W, Green-Thompson ZW, Nixon RA, Saksida LM, Bussey TJ, O’Kane CJ, Rubinsztein DC (2015). Calpain inhibition mediates autophagy-dependent protection against polyglutamine toxicity. Cell Death Differ.

[54] Herenger Y, Stoetzel C, Schaefer E, Scheidecker S, Manière MC, Pelletier V, Alembik Y, Christmann D, Clavert JM, Terzic J, Fischbach M, De Saint Martin A, Dollfus H (2015). Long term follow up of two independent patients with schinzel-giedion carrying SETBP1 mutations. Eur J Med Genet.

[55] Ko JM, Lim BC, Kim KJ, Hwang YS, Ryu HW, Lee JH, Kim JS, Chae JH (2013). Distinct neurological features in a patient with schinzel-giedion syndrome caused by a recurrent SETBP1 mutation. Childs Nerv Syst ChNS Off J Int Soc Pediatr Neurosurg.

[56] Diguet E, Fernagut PO, Normand E, Centelles L, Mulle C, Tison F (2004). Experimental basis for the putative role of GluR6/kainate glutamate receptor subunit in huntington’s disease natural history. Neurobiol Dis.

[57] Jarabek BR, Yasuda RP, Wolfe BB (2004). Regulation of proteins affecting NMDA receptor-induced excitotoxicity in a huntington’s mouse model. Brain.

[58] Smith AK, Fang H, Whistler T, Unger ER, Rajeevan MS (2011). Convergent genomic studies identify association of GRIK2 and NPAS2 with chronic fatigue syndrome. Neuropsychobiology.

[59] Hyndman KA, Boesen EI, Elmarakby AA, Brands MW, Huang P, Kohan DE, Pollock DM, Pollock JS (2013). Renal collecting duct NOS1 maintains fluid-electrolyte homeostasis and blood pressure. Hypertension.

[60] Lee KA, Hammerle LP, Andrews PS, Stokes MP, Mustelin T, Silva JC, Black RA, Doedens JR (2011). Ubiquitin ligase substrate identification through quantitative proteomics at both the protein and peptide levels. J Biol Chem.

[61] Kim W, Bennett EJ, Huttlin EL, Guo A, Li J, Possemato A, Sowa ME, Rad R, Rush J, Comb MJ, Harper JW, Gygi SP (2011). Systematic and quantitative assessment of the ubiquitin-modified proteome. Mol Cell.

[62] Niikura T, Hashimoto Y, Tajima H, Ishizaka M, Yamagishi Y, Kawasumi M, Nawa M, Terashita K, Aiso S, Nishimoto I (2003). A tripartite motif protein TRIM11 binds and destabilizes humanin, a neuroprotective peptide against alzheimer’s disease-relevant insults. Eur J NeuroSci.

[63] Peng HM, Morishima Y, Jenkins GJ, Dunbar AY, Lau M, Patterson C, Pratt WB, Osawa Y (2004). Ubiquitylation of neuronal nitric-oxide synthase by CHIP, a chaperone-dependent e3 ligase. J Biol Chem.

[64] Wagner SA, Beli P, Weinert BT, Nielsen ML, Cox J, Mann M, Choudhary C (2011). A proteome-wide, quantitative survey of in vivo ubiquitylation sites reveals widespread regulatory roles. Mol Cell Proteomics MCP.

[65] Steffan JS, Agrawal N, Pallos J, Rockabrand E, Trotman LC, Slepko N, Illes K, Lukacsovich T, Zhu YZ, Cattaneo E, Pandolfi PP, Thompson LM, Marsh JL (2004). SUMO modification of huntingtin and huntington’s disease pathology. Science (New York, N.Y.).

[66] Kaltenbach LS, Romero E, Becklin RR, Chettier R, Bell R, Phansalkar A, Strand A, Torcassi C, Savage J, Hurlburt A, Cha GH, Ukani L, Chepanoske CL, Zhen Y, Sahasrabudhe S, Olson J, Kurschner C, Ellerby LM, Peltier JM, Botas J, Hughes RE (2007). Huntingtin interacting proteins are genetic modifiers of neurodegeneration. PLoS Genet.

[67] Harjes P, Wanker EE (2003). The hunt for huntingtin function: interaction partners tell many different stories. Trends Biochem Sci.

[68] Carroll JB, Bates GP, Steffan J, Saft C, Tabrizi SJ (2015). Treating the whole body in huntington’s disease. Lancet Neurol.

