# Supplementary material for: Common disease signatures from gene expression analysis in Huntington’s disease human blood and brain
Source: Orphanet J Rare Dis. 2016 Aug 1;11:97. doi: 10.1186/s13023-016-0475-2 (PMC4968014; doi:10.1186/s13023-016-0475-2)
Supplement: Additional file 1 — Significantly correlated modules in brain. This file contains the modules from each brain region (caudate, BA4, BA9 and cerebellum) that were associated with the disease phenotype. The numbers (P v a l u e) indicate the correlation of each module with the disease phenotype. Green: negative correlation, red: positive correlation. The intensity of the color depicts the strength of the correlation. (PDF 1341 kb) [file 13023_2016_475_MOESM1_ESM.pdf]

WGCNA in brain dataset

Significantly correlated modules from caudate, cerebellum, BA4 and BA9 to the disease phenotypes

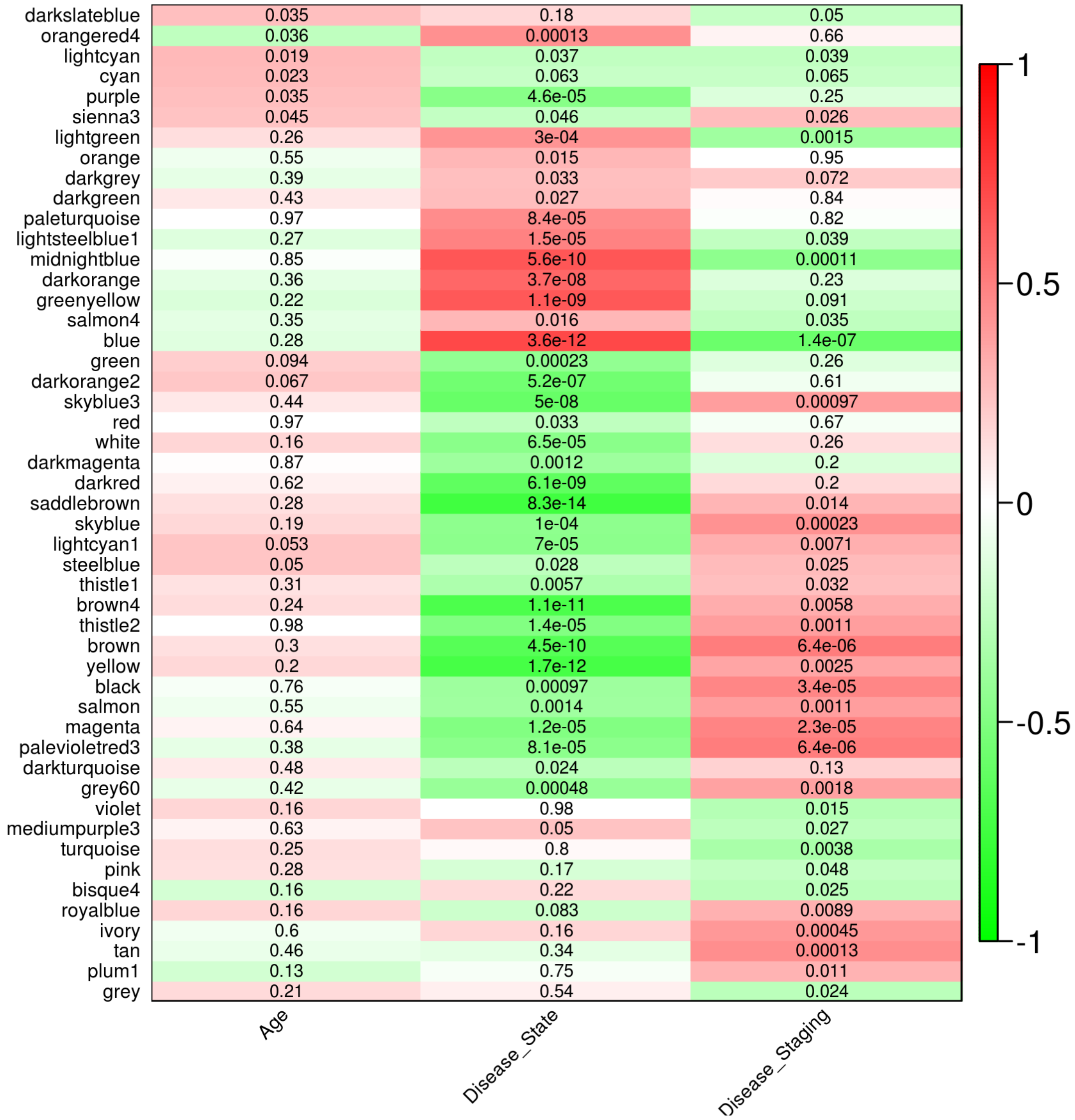

Figure 1 Significantly correlated modules from the caudate nucleus

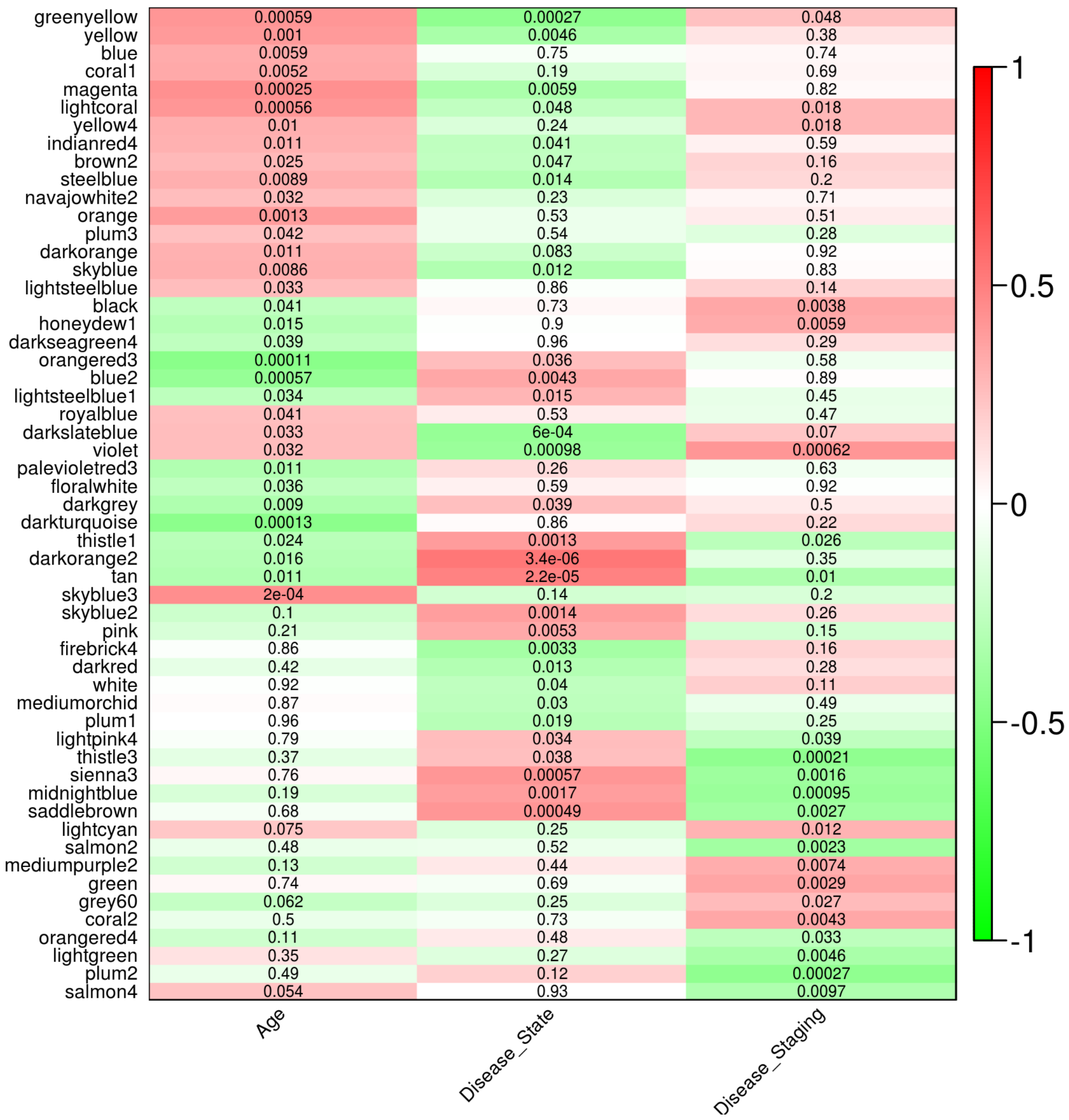

Figure 2 Significantly correlated modules from the cerebellum

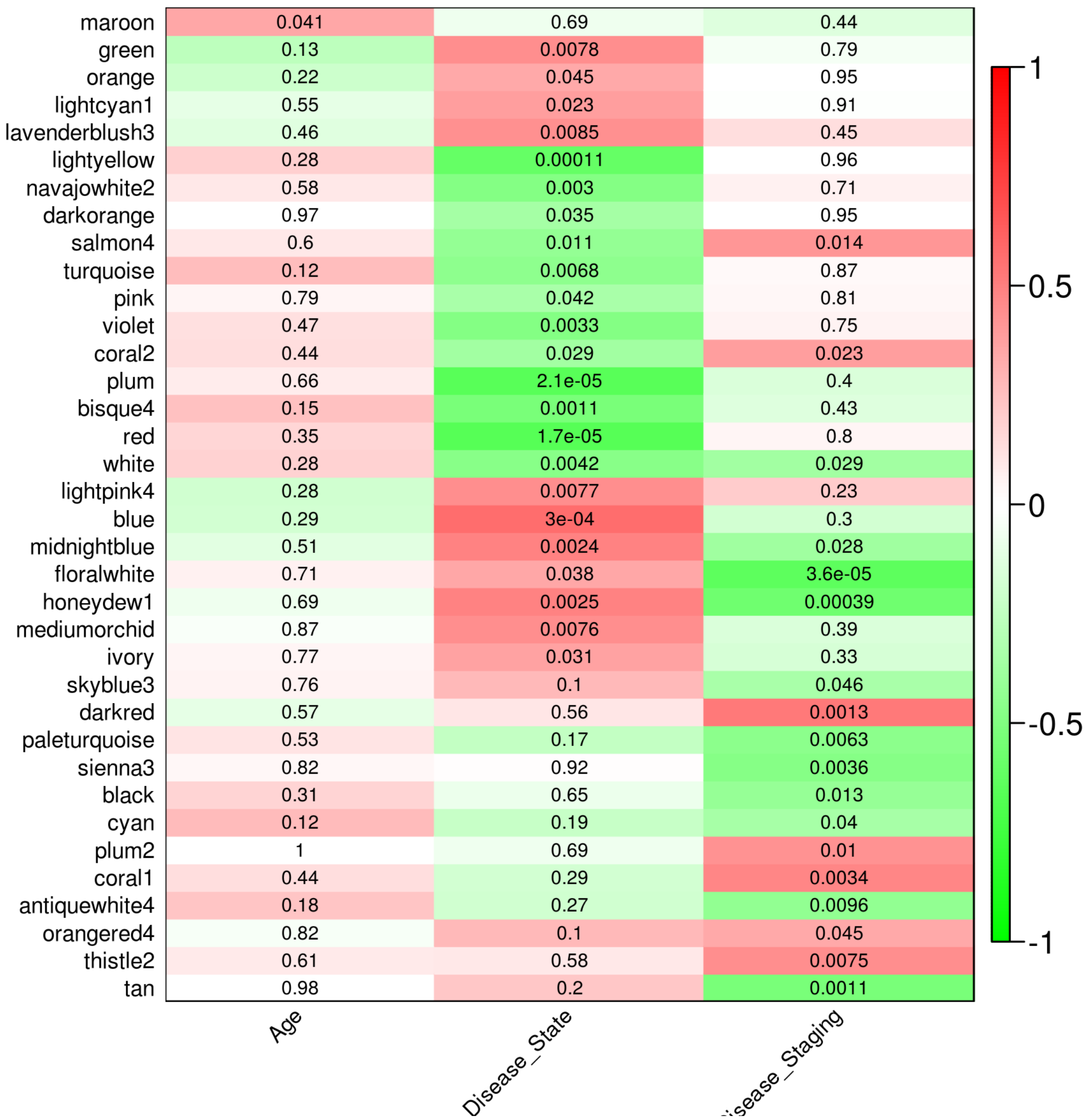

Figure 3 Significantly correlated modules from the BA4

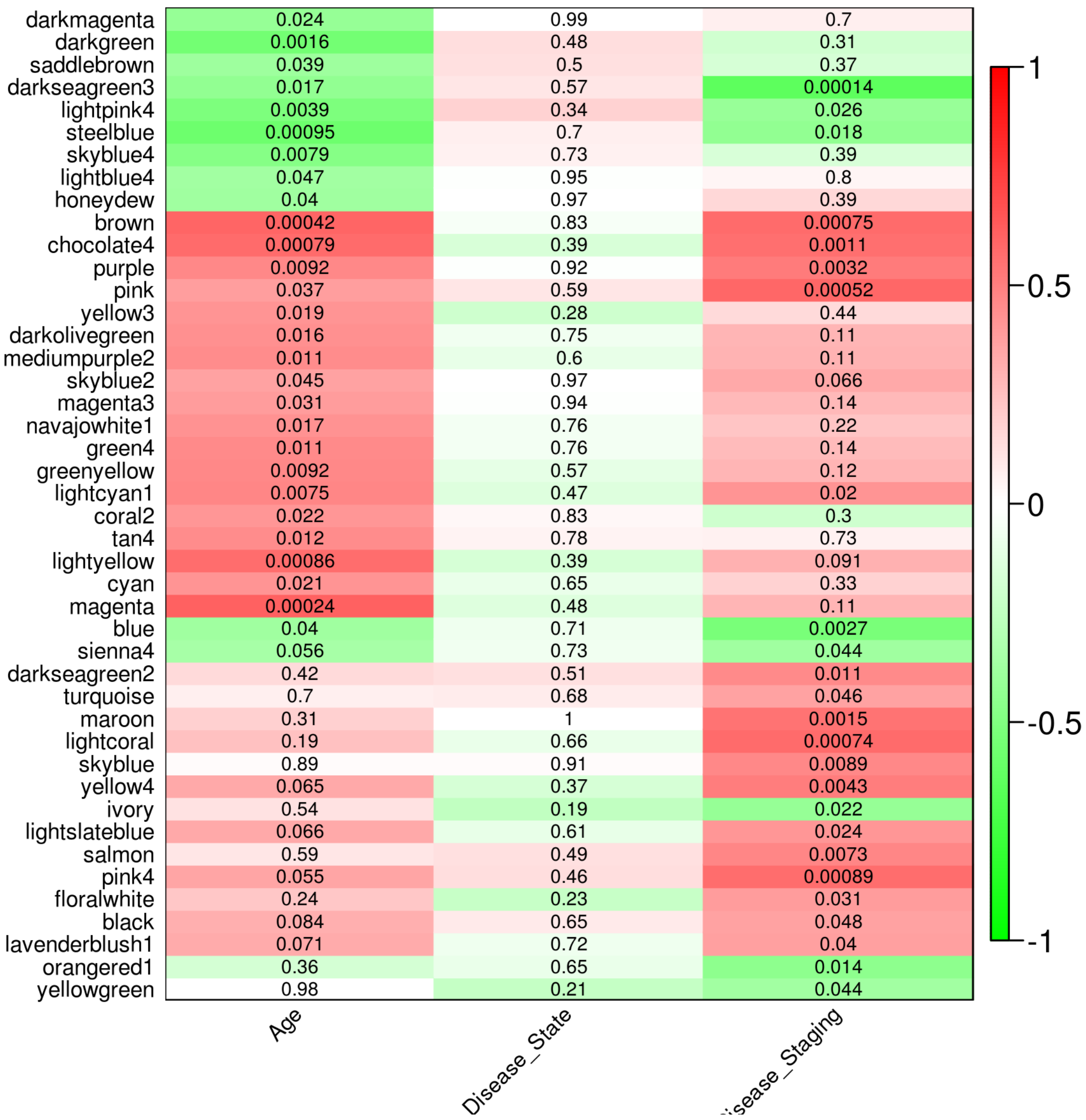

Figure 4 Significantly correlated modules from the BA9
