# Supplementary figures and images for: Common disease signatures from gene expression analysis in Huntington’s disease human blood and brain
Source: Orphanet J Rare Dis. 2016 Aug 1;11:97. doi: 10.1186/s13023-016-0475-2 (PMC4968014; doi:10.1186/s13023-016-0475-2)

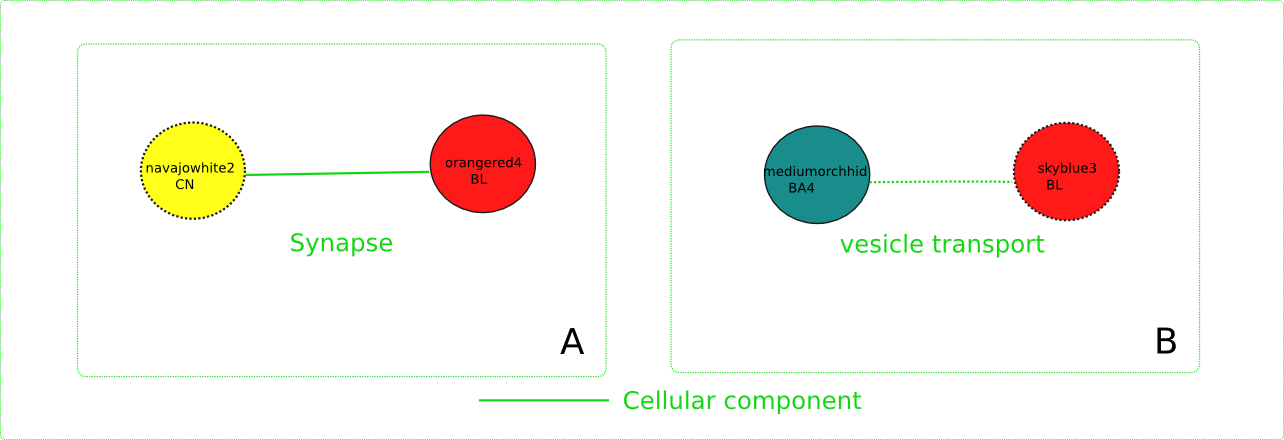

Supplement: Additional file 2 — Additional disease signatures. This file contains the additional disease signatures that were identified with less stringent statistical criteria. The yellow circle indicates a caudate nucleus module, bluegrey circle indicates a module from the BA4 brain region and red color indicates blood modules. The line between two modules indicates a significant link (blood-brain pair) between modules (F W E R<=10 %). Dashed line indicates a module pair with marginal significance (F W E R<50 %) while dashed circles indicate modules with marginal significance with the disease phenotype. The green line between module pairs defines an association based on cellular component. (PNG 66.6 kb) [file 13023_2016_475_MOESM2_ESM.png]

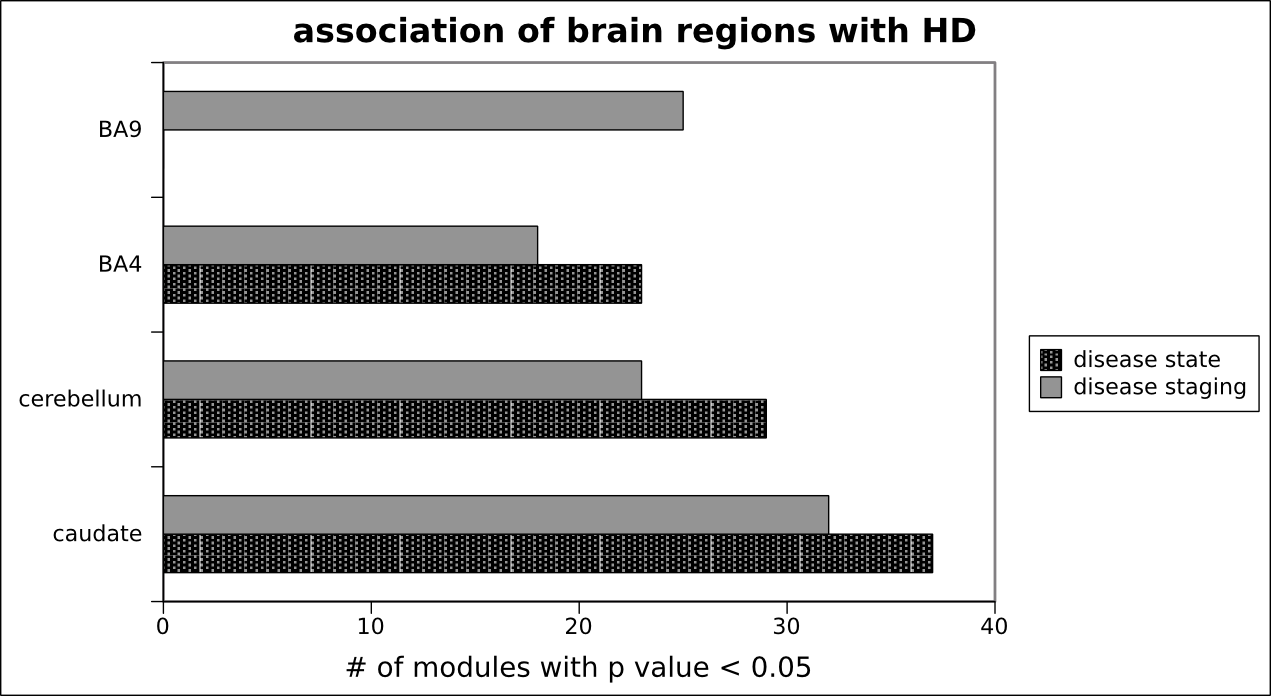

Supplement: Additional file 6 — Association of the brain regions with the disease phenotype. This file describes the total number of modules, per brain region, that are associated with the the two HD phenotypes; the disease state (HD or control) and disease staging (grade 0 - 5) as that was resulted by the WGCNA. (PNG 54.9 kb) [file 13023_2016_475_MOESM6_ESM.png]
