# Supplementary material for: Common disease signatures from gene expression analysis in Huntington’s disease human blood and brain
Source: Orphanet J Rare Dis. 2016 Aug 1;11:97. doi: 10.1186/s13023-016-0475-2 (PMC4968014; doi:10.1186/s13023-016-0475-2)
Supplement: Additional file 3 — Modules in blood significantly correlated with HD. This file contains the 8 modules that were identified in blood as being correlated to the HD phenotype. The file describes each module according to the most representative annotations per semantic category (biological processes, cellular component, molecular function, disease or syndrome). (DOC 16.5 kb) [file 13023_2016_475_MOESM3_ESM.doc]

|  | Biological processes | Cellular component | Molecular function | Disease or syndrome |
| --- | --- | --- | --- | --- |
| *blue | Sumoylation,  Nuclear Export,  chromatin modification | endosome, ribonucleoprotein complex location | ubiquitin activity, protein kinase activity, ubiquitin activity | Neurodegenerative Disorders (HD,AD), retinitis pigmentosa |
| salmon4 | lipid transport, spliceosome, rna splicing, gtpase related activity | snrnp location,  spliceosome | enzyme & protein activity,transporter activity | Cholestasis, cataract |
| *violet | immune response | interleukin complex, integral to membrane, cytolytic granule | interleukin binding activity | viral infections, lymphocyte related disorders, autoimmune disorders |
| *green | cell-cycle, protein modification | endosome, golgi network | kinase activity, ubiquitin activity | Ataxia, tuberous sclerosis 1 &2, spastic paraplegia |
| plum2 | developmental processes, cell-cycle, protein ubiquitination | septins, b-cell receptor | ubiquitin activity, enzyme activity (vetispiradiene, ligase) | herpesvirus infection, developmental disorders |
| orangered4 | cell signaling, synaptic transmission | synapses | glutamate activity, nitric oxide synthase activity | epilepsy, ataxia, diabetic neuropathy |
| *plum1 | meiosis, mitosis | golgi network, endosome | cholesterol activity, enzyme activity | SCAs |
| thistle2 | sphingolipid biosynthetic processes, regulation of actin filament | Actin, filaments | serine activity, kinase activity | muscular dystrophy, sensory neuropathy |

Table 1:
